# Supplementary material for: The Aspergillus fumigatus Extracellular Polysaccharide Galactosaminogalactan Displays Context-Dependent Cooperative and Competitive Social Traits in Mixed Biofilms
Source: J Fungi (Basel). 2025 Sep 25;11(10):695. doi: 10.3390/jof11100695 (PMC12564968; doi:10.3390/jof11100695)
Supplement: Supplementary file 1 [file jof-11-00695-s001.zip › jof-3882496-supplementary.pdf]

Supplementary Material

Supplementary Figures

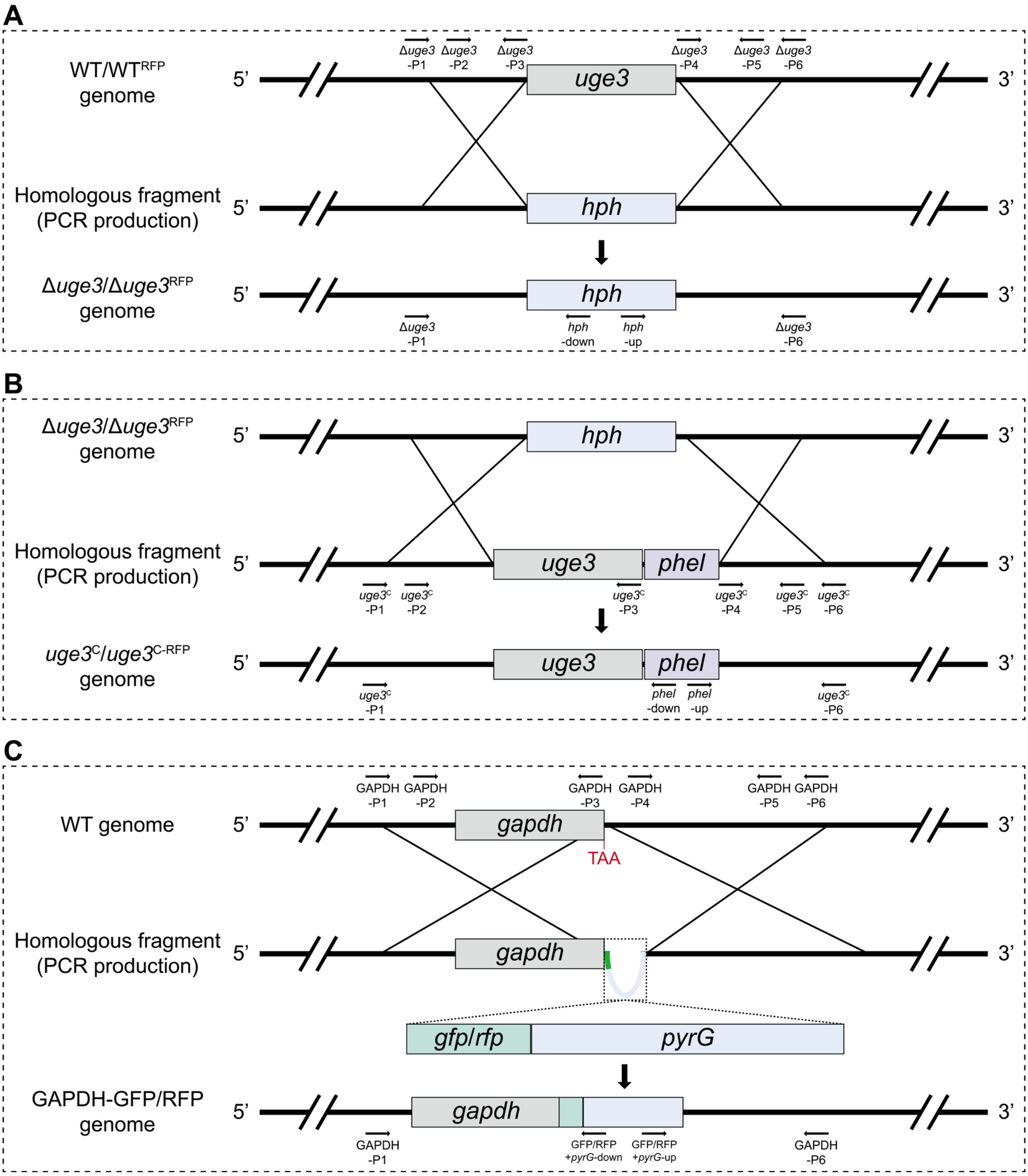

Supplementary Figure S1. The schematic diagram of the construction of the indicated strains (A) Δ*uge3* and Δ*uge3*^{RFP}, (B) *uge3*^{C} and *uge3*$^{C\text{-}RFP}$, (C) WT^{GFP} and WT^{RFP}.

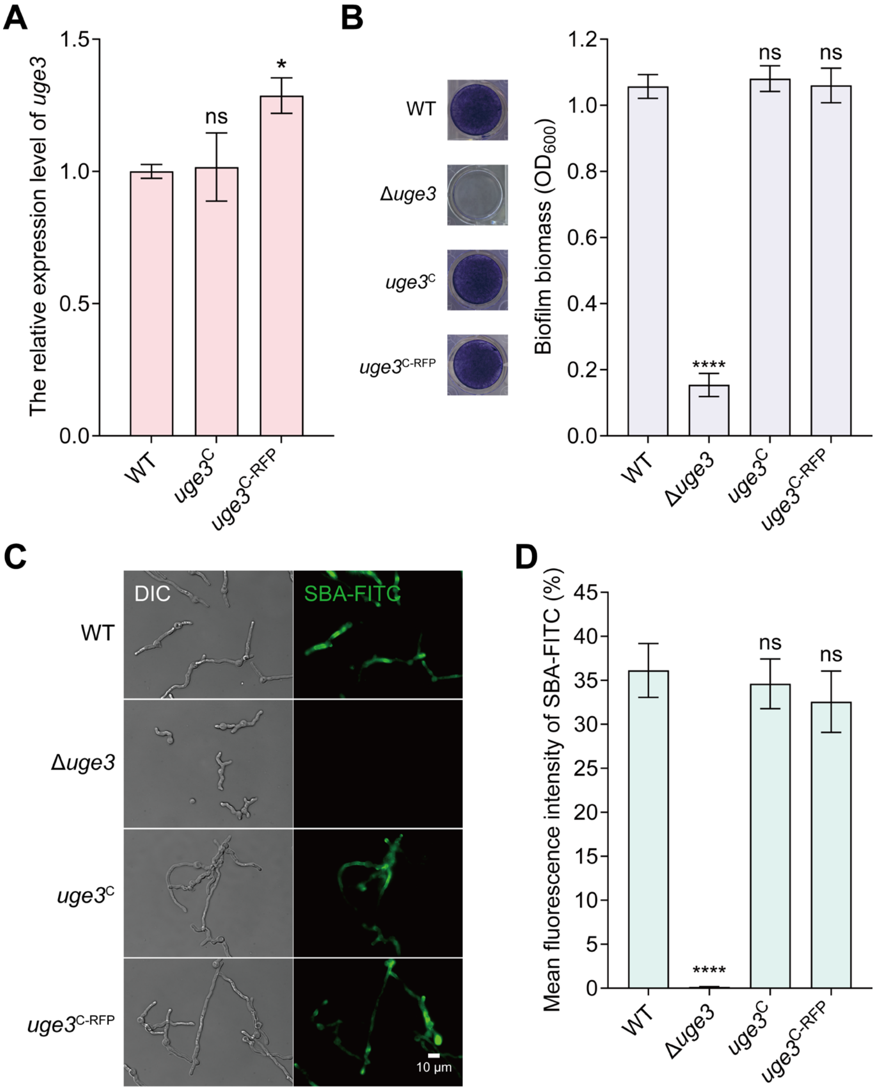

Supplementary Figure S2. The verification of complemented strains *uge3*^{C} and *uge3*$^{C\text{-}RFP}$.
(**A**) The relative transcript levels of *uge3* in indicated strains. Conidia of WT, *uge3*^{C} and *uge3*$^{C\text{-}RFP}$ were grown at 220 rpm and 37°C for 24 hours in minimal medium (MM). The mRNA levels were normalized to the reference gene *tubA*. (**B**) Representative crystal violet assay photos (left panels) and biomass (right panels) of indicated strains biofilms. Conidia of WT, Δ*uge3* mutant, *uge3*^{C} and *uge3*$^{C\text{-}RFP}$ were statically grown for 24 hours in MM. The biofilm biomass was determined by a crystal violet assay. (**C**) Representative images of the indicated strains stained with a GAG-specific fluorescein-tagged soybean agglutinin lectin (SBA-FITC). Conidia of WT, Δ*uge3* mutant, *uge3*^{C} and *uge3*$^{C\text{-}RFP}$ were statically grown for 9 hours in MM. (**D**) Quantification of SBA-FITC fluorescence intensity in (C). Experiments were conducted a minimum of three times, with each bar indicating the mean ± standard deviation (SD). A one-way analysis of variance (ANOVA) along with multiple comparison tests was utilized for statistical analysis. *$P<0.05$; ****$P<0.0001$; ns represents no significance.

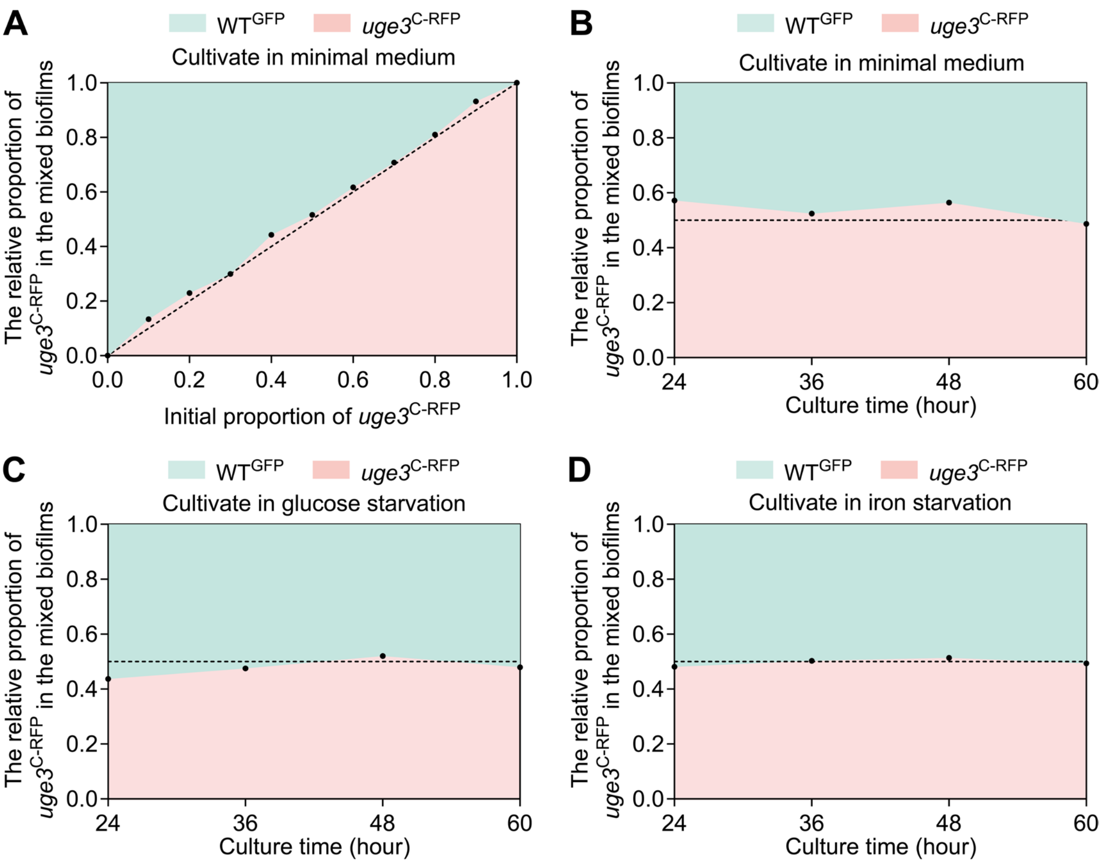

Supplementary Figure S3. Complemented strain *uge3*$^{\mathbf{C\text{-}RFP}}$ displayed nearly identical fitness to WT$^{\mathbf{GFP}}$ at all tested initial proportions and nutrient conditions.

(**A**) The relative fitness of GAG$^+$ (WT^{GFP}) and GAG$^+$ (*uge3*$^{C\text{-}RFP}$) in the mixed biofilms at different ratios. Conidia of WT^{GFP} and *uge3*$^{C\text{-}RFP}$ at indicated ratios were statically grown for 24 hours in MM. (**B**) The relative fitness of GAG$^+$ (WT^{GFP}) and GAG$^+$ (*uge3*$^{C\text{-}RFP}$) in the mixed biofilms. Conidia of WT^{GFP} and *uge3*$^{C\text{-}RFP}$ at a 1:1 ratio were statically grown in MM and biofilms were collected at indicated time points. (**C** and **D**) The relative fitness of GAG$^+$ (WT^{GFP}) and GAG$^+$ (*uge3*$^{C\text{-}RFP}$) in the mixed biofilms under nutrient limitation. Conidia of WT^{GFP} and *uge3*$^{C\text{-}RFP}$ at a 1:1 ratio were statically grown in glucose starvation medium (MM containing 0.05% glucose) (C) and iron starvation medium (MM without addition of iron) (D), biofilms were collected at indicated time points. The relative proportion of *uge3*$^{C\text{-}RFP}$ in the mixed biofilms was quantitated by quantitative PCR.

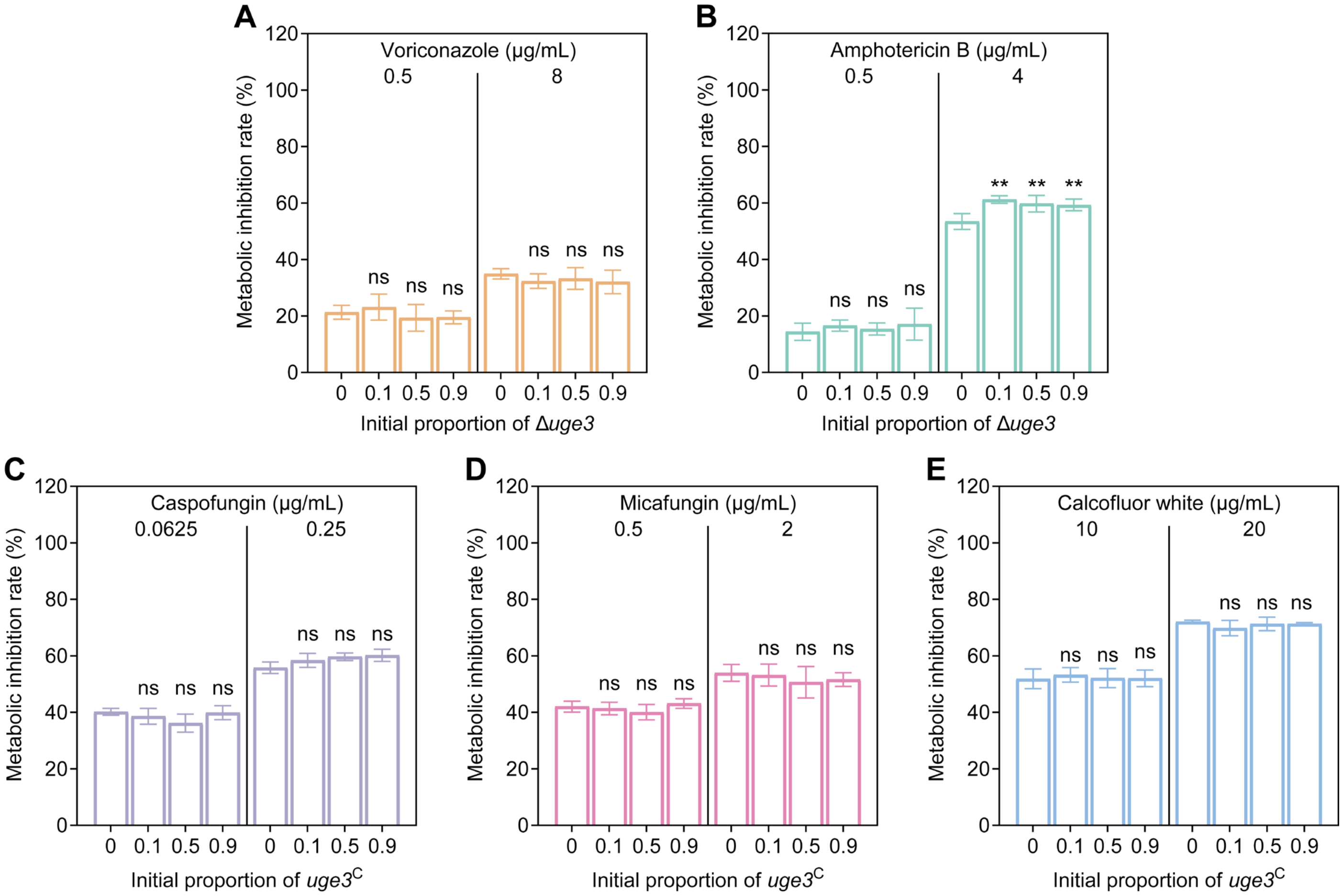

Supplementary Figure S4. The metabolic inhibition assay of mixed biofilms.

(**A** and **B**) Conidia of WT and Δ*uge3* mutant at indicated ratios were statically grown for 24 hours in MM and then treated with voriconazole (A) and amphotericin B (B) at the indicated concentrations for an additional 12 hours. (**C** – **E**) Conidia of WT and *uge3*^{C} at indicated ratios were statically grown for 24 hours in MM and then treated with caspofungin (C), micafungin (D) and calcofluor white (E) at the indicated concentrations for an additional 12 hours. The metabolic activity after treatments was determined by XTT assay and the percentage of metabolic inhibition compared with non-treated cells. Experiments were conducted a minimum of three times, with each bar indicating the mean ± standard deviation (SD). A one-way analysis of variance (ANOVA) along with multiple comparison tests was utilized for statistical analysis. **$P<0.01$; ns represents no significance.

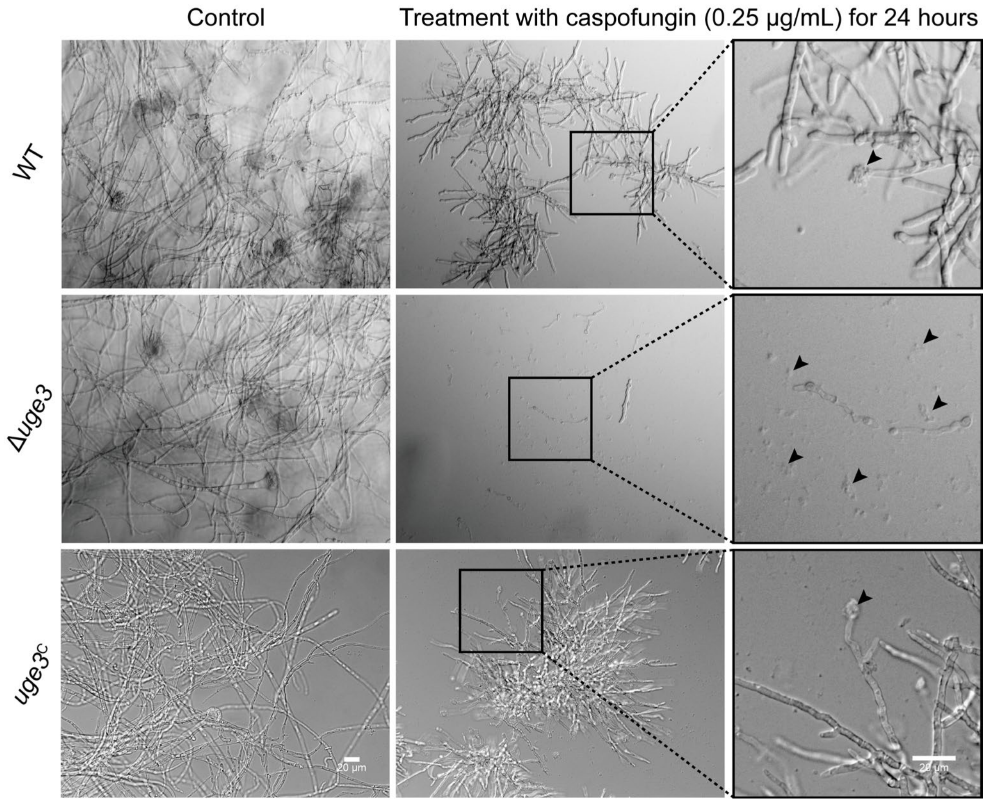

Supplementary Figure S5. Representative images of WT, Δ*uge3* mutant and *uge3*^{C} hyphae grown under treated with caspofungin.

Conidia of WT, Δ*uge3* mutant and *uge3*^{C} were separately grown for 10 hours in MM, then treated with caspofungin for an additional 24 hours. The right panels show magnifications of the framed sections in the left panels. Examples of lysed germlings are indicated with black arrowheads.

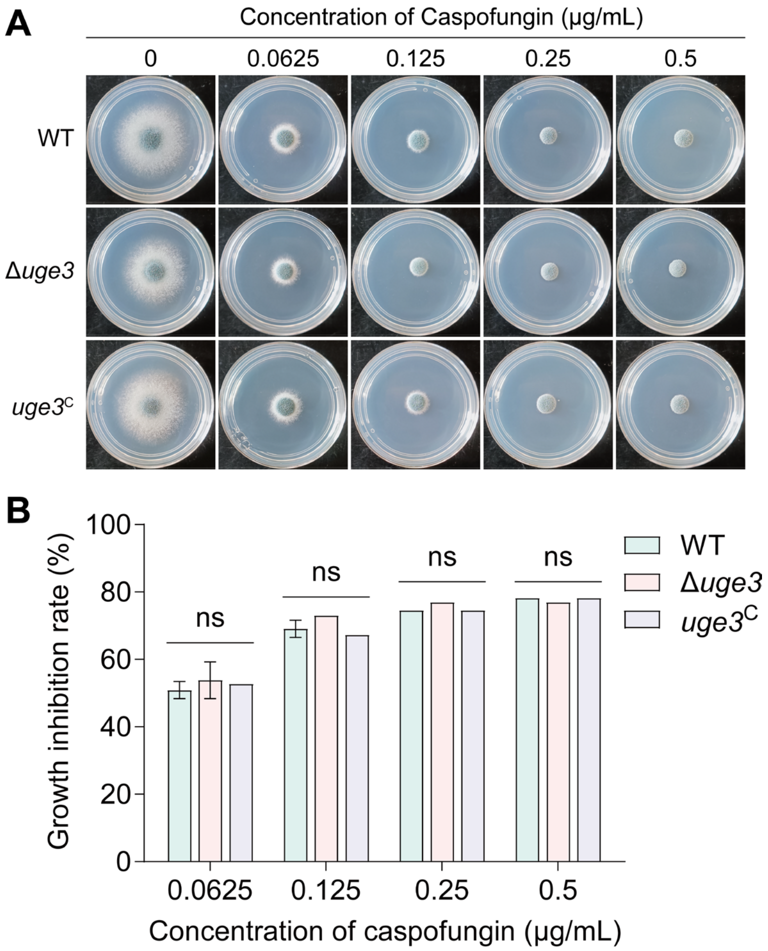

Supplementary Figure S6. Sensitivity test of WT, Δ*uge3* mutant and *uge3*^{C} to caspofungin on agar plates.

(**A**) Colony growth of WT, Δ*uge3* mutant and *uge3*^{C} on MM with or without caspofungin for 48 hours. Agar plates were inoculated with 2×10^4 conidia. (**B**) Growth inhibition rate of WT, Δ*uge3* mutant and *uge3*^{C} on MM with or without caspofungin for 48 hours. Quantitative analysis of colony diameter in (A) was conducted using the growth inhibition rate. Caspofungin-treated colony diameters were compared to untreated colony diameters to calculate the inhibition of growth. Statistical analysis was performed using one-tailed, unpaired *t*-tests. ns represents no significance.

Supplementary Tables

Supplementary Table S1. Strains used in this study

Strains	Genotype	Reference
AF1160	Δ*ku80*, *pyrG*	FGSC
WT (AF1161)	AF1160::*pyrG*	[30]
Δ*uge3*	AF1161, Δ*uge3*::*hph*	This study
WT^{GFP}	AF1160, *gapdh*::*gfp*::*pyrG*	This study
WT^{RFP}	AF1160, *gapdh*::r*fp*::*pyrG*	This study
Δ*uge3*^{RFP}	WT^{RFP}, Δ*uge3*::*hph*	This study
uge3^{C}	Δ*uge3*, Δ*hph*::*uge3*::*phel*	This study
uge3$^{C\text{-}RFP}$	Δ*uge3*^{RFP}, Δ*hph*::*uge3*::*phel*	This study

Supplementary Table S2. Primers used in this study

Name/Purpose	Sequence (5′ to 3′)
**Genes deletion and confirmation**	
Δ*uge3*-P1	CACGACAGCCAGAATAGC
Δ*uge3*-P2	TCAAGGGGAAACGCATCAAG
Δ*uge3*-P3	GCACCGGTCAACCATGATCTACTCGGACAGATTGAGAACC
Δ*uge3*-P4	ATGGTTGCCTAGTGAATGCTCCGGCTCATCCTGTTCTTTCTTGG
Δ*uge3*-P5	ATGCGTTCTGACTTGTTGC
Δ*uge3*-P6	TCGTCATCTCCTCGCAGTG
Δ*uge3*-SF	CGACAATCTCAGCAACTCC
Δ*uge3*-SR	CCCGTCCTCAGTATTCCA
hph-F	AGATCATGGTTGACCGGTGC
hph-R	CGGAGCATTCACTAGGCAACCAT
hph-down	GTCACTGTACAGAGCTCACG
hph-up	GTTGGTGTCGATGTCAGCTC
**Complemented strains construction and confirmation**	
uge3^{C}-P1	GTCCAAGTCAGCCCTAGATAC
uge3^{C}-P2	TGATGTGATCTGGTCCGG
uge3^{C}-P3	TAATCAATTGCCCGTCTGTCACAGGCCGCTCGATATTACG
uge3^{C}-P4	GCTTACATTCACGCCCTCCTGTCGGCCCATAATATATGCAAG
uge3^{C}-P5	ACGCAAGAATGTGTACAGTGC
uge3^{C}-P6	CAGGCATTGATGCTCTTGC
phel-F	TGACAGACGGGCAATTGATTA
phel-R	AGGAGGGCGTGAATGTAAGC
phel-down	CCATGACTTCCATCGTATGCC
phel-up	ACGACGTGACCCTGTTCATC
**Reporter strains construction and confirmation**	
GAPDH-P1	TTGGTATCAACGGCTTCGGTCG
GAPDH-P2	CGAGTAATCGCAGGTCTTCC

GAPDH-P3	CCAGCGCCTGCACCAGCTCCCTGGGAATCGACCTTGGCGA
GAPDH-P4	CATCAGTGCCTCCTCTCAGACAGTAGGAATCAGGACGGAAAAT
GAPDH-P5	GTCACCATCCTGCTCCATAG
GAPDH-P6	GCAGAGGCAGATTCATAGAC
GFP+*pyrG*-F	GGAGCTGGTGCAGGCGCTGG
GFP+*pyrG*-R	CTGTCTGAGAGGAGGCACTGATG
GFP+*pyrG*-down	GTGAAGAGCATTGTTTGAGGC
GFP+*pyrG*-up	GATACAGGTCTCGGTCCCTA
RFP+*pyrG*-F	GGAGCTGGTGCAGGCGCTGG
RFP+*pyrG*-R	CTGTCTGAGAGGAGGCACTGATG
RFP+*pyrG*-down	GTGAAGAGCATTGTTTGAGGC
RFP+*pyrG*-up	GATACAGGTCTCGGTCCCTA
**Quantitative PCR**	
qPCR-GFP-F	ACGACGGGAACTACAAGACACG
qPCR-GFP-R	TGTGGCCGAGGATGTTTCCG
qPCR-RFP-F	CTTCAAGTGGGAGCGCGTGA
qPCR-RFP-R	GGTACATCCGCTCGGTGGAG
qPCR-*uge3*-F	GCTGTTAGCCTCCCAGTACC
qPCR-*uge3*-R	GGACTTGGTCGTACCCCAT
qPCR-*tubA*-F	TCCCAACAACATCCAGACCG
qPCR-*tubA*-R	TGATCACCGACACGCTTGAA
